# Missed Opportunities: Evolution of Patients Leaving without Being Seen or against Medical Advice during a Six-Year Period in a Swiss Tertiary Hospital Emergency Department

**DOI:** 10.1155/2014/690368

**Published:** 2014-06-12

**Authors:** Pierre-Nicolas Carron, Bertrand Yersin, Lionel Trueb, Philippe Gonin, Olivier Hugli

**Affiliations:** Emergency Department, Lausanne University Hospital, 1011 Lausanne, Switzerland

## Abstract

*Aim.* The study aimed at describing the evolution over a 6-year period of patients leaving the emergency department (ED) before being seen (“left without being seen” or LWBS) or against medical advice (“left against medical advice” or LAMA) and at describing their characteristics. *Methods.* A retrospective database analysis of all adult patients who are admitted to the ED, between 2005 and 2010, and who left before being evaluated or against medical advice, in a tertiary university hospital. *Results.* During the study period, among the 307,716 patients who were registered in the ED, 1,157 LWBS (0.4%) and 1,853 LAMA (0.9%) patients were identified. These proportions remained stable over the period. The patients had an average age of 38.5 ± 15.9 years for LWBS and 41.9 ± 17.4 years for LAMA. The median time spent in the ED before leaving was 102.4 minutes for the LWBS patients and 226 minutes for LAMA patients. The most frequent reason for LAMA was related to the excessive length of stay. *Conclusion.* The rates of LWBS and LAMA patients were low and remained stable. The patients shared similar characteristics and reasons for leaving were largely related to the length of stay or waiting time.

## 1. Introduction

In most western hospitals, a certain number of patients leave the emergency department (ED) before receiving a full medical evaluation “incomplete emergency care.” The literature distinguishes between two types of such cases as follows.

- Patients who leave before being seen by a physician (“*left without being seen*” or LWBS). This refers to patients who leave the ED from the waiting room, after having completed their administrative paperwork and usually an initial evaluation by a triage nurse (TN). The reported rate of LWBS patients is between 0.1% and 15% of consultations to the ED, depending on the type and size of the hospital [1–14].
- Patients who leave the ER against medical advice (“*left against medical advice*” or LAMA) during their ED stay, either during the diagnostic period (while awaiting an X-ray exam, a specialized consultation, etc.) or during the treatment process (refusal of treatment or hospitalization). The annual rate of LAMA patients is estimated at 0.5–3% of admissions in the ED [2, 15–20].

These two populations are reported to share similar sociodemographic characteristics and identifying factors, such as triage level, initial complaints, or insurance coverage [2]. They are often analyzed in parallel and are considered as “missed opportunities” for the ED and healthcare system [2]. LWBS and LAMA patients are both related to the length of stay or waiting times and are therefore used as indirect indicators of ED overcrowding and of the quality of ED care [7, 12, 13].

Most ED studies concerning LWBS and LAMA patients were conducted in North America or Australia, with a majority of cross-sectional analyses [1, 2, 6, 13, 21]. In Europe, only a limited number of studies have been conducted [4, 22]. In order to get a better picture of the evolution over time of these “missed opportunities,” a retrospective study of patients leaving the ED of a Swiss tertiary university hospital before being seen or against medical advice was conducted for the period 2005–2010. The goal of this study was to evaluate the rate and evolution over time of adult patients who left the ED without being seen (LWBS) or against medical advice (LAMA) and to identify the demographic and medical characteristics of these patients, as well as the contextual elements related to these departures (day, time, and reason for leaving).

## 2. Patients and Methods

### 2.1. Setting

This is a retrospective study of all adult patients (≥16 years) who are admitted to the ED of Lausanne University Hospital between 2005 and 2010 and who left before being evaluated by a physician or against medical advice.

This Swiss hospital has 1,400 acute beds and serves as the local primary care hospital as well as a tertiary university-based teaching hospital. The ED receives approximately 50,000 adult patients per year, initially evaluated by triage nurses (TN). Many patients are sent for specialized consultations (ophthalmology, gynecology, psychiatry, etc.) or to an ambulatory primary care clinic and only 35,000 patients are ultimately admitted and treated in the ED. Pediatric patients are treated at another hospital site and only life-threatening pediatric emergencies are admitted to our center. Our ED has a 25-bed short-stay unit, where patients are either kept under observation until discharge or boarded while awaiting a hospital bed.

Patients admitted to the ED are initially registered in the ED software and evaluated by a TN. They are triaged according to the reasons for the visit and their vital signs. They are then registered in the administrative information system unless their clinical condition warrants immediate attention. Each patient is assigned one triage category based on the presumed urgency of the case. Up until the end of 2009, the 5-level Lausanne triage scale (LTS) was used. In 2010, it was replaced with the four-level Swiss triage scale (STS) [23]. Triage categories 1 (patient requiring immediate attention and evaluation) and 2 (patient requiring evaluation within 20 minutes) are considered as urgent. These two categories were not affected by the changes in triage scale. Semiurgent category 3 (patient requiring evaluation within 120 minutes) and category 4 (nonurgent conditions) complete the STS.

### 2.2. Patients

According to the international and national definitions, the patients were divided into two categories as follows.

1. LWBS patients: adult patients (≥16 years) who came to the ED, were evaluated and triaged by a TN usually, but left the ED before being evaluated by a physician.
2. LAMA patients: adult patients (≥16 years) admitted in the ED and evaluated by a physician, and who left against medical advice prior to the physician's final evaluation and/or treatment.

### 2.3. Data Collection

All patients who left prior to being evaluated or who left against medical advice from 2005 to 2010 were included according to the information in the administrative patient management system, based on two complementary databases (AXYA database for the institutional administrative management system and Gyroflux database for the ED patients flow software), and in the dedicated medical charts. Administrative data mentioned in this study is used for quality and financial performance controls and is thus considered exhaustive. Volumes for ED visits represent the actual number of ED stays. Overall 98% of ED visits registered in the two databases could be matched through patients' personal unique identifier and hospital stay unique identifier.

In the event of LAMA, internal rules mandate that there should be a medical contact and a signature of a specific institutional form by the patient, the nurse, and the physician in charge. This form indicates the time of departure, any pertinent clinical elements, and reason cited for leaving, as well as possible recommendations and advice given to the patient. In case of LBWS, the same form is systematically used to document the patient's departure and time of departure (or time at which the triage nurse realized the patient had left). The stated reason for leaving was hardly ever documented in LBWS patients and was therefore not documented. The forms for LWBS and LAMA patients are systematically collected and are part of a continuous review process by one of the senior physicians of the ED. The administrative data (time of arrival) and deciding factors of the TN (reason for refusal and triage category) are part of the database of the ER patient management system as well as the administrative patient management system.

The following data were collected: age, gender, time and date of the index visit, initial complaint, initial triage category, according to the Swiss triage scale, cited reason for departure, and length of stay in the ED before leaving, according to the information in the administrative patient management system and in the forms for LWBS or LAMA. The missing information was completed using the nursing and medical records. Missing data were not imputed.

### 2.4. Statistical Analysis

All data were deidentified and transferred into a computerized database (Microsoft Access, Microsoft Corp., Redmond, WA). Categorical data are presented as counts and percentages and continuous variables as mean ± standard deviation or median with their interquartile range. Statistical analysis was performed using Stata Statistical Software Release 12.0 (Stata Corporation, College Station, TX).

### 2.5. Ethical Consideration

Because of the retrospective nature of this research and its anonymity, the study did not require personal information or explicit agreement of the patients. The study received approvals from the local Institutional Ethics Committee.

## 3. Results 

From January 1, 2005, to December 31, 2010, a total of 307,716 patients were registered and triaged in the ED. A total of 3,027 LWBS or LAMA forms were collected. After excluding the duplicates, pediatric cases, and errors (leaving with medical consent or following a complete medical evaluation), 3,010 forms (99.4%) were included for analysis, representing 0.98% of the total number of triaged patients. 1,157 LWBS patients were identified (0.4%), with a rate that remained stable over the six-year period of the study (Table 1).

One-third of the patients registered and triaged in the ED were referred to outside consultations and a total of only 202,551 patients were ultimately evaluated in the ED. 1,853 of these latter patients left the ED against medical advice, representing an average of 0.9% of all patients that were seen (Table 2). This rate of LAMA was also stable over the entire study period (*P* > 0.05).

**Figure 1:**
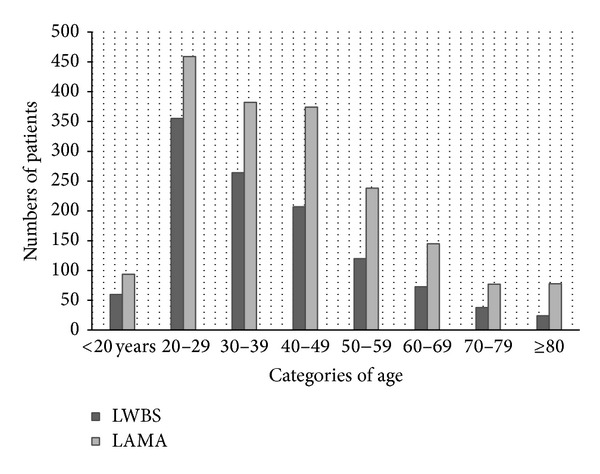
Numbers of LWBS and LAMA patients according to age.

The gender distribution shows a slight predominance of men in both groups (statistically insignificant), except for the LWBS patients in 2009. Men comprised 632 cases (54.6%) of the LWBS group and 1015 (54.8%) of the LAMA group. The patients had an average age of 38.5 ± 15.9 years for LWBS and 41.9 ± 17.4 years for LAMA (*P* < 0.05, Figure 1).

The proportion of LMA and LWBS shows no significant daily or seasonal variations.

**Figure 2:**
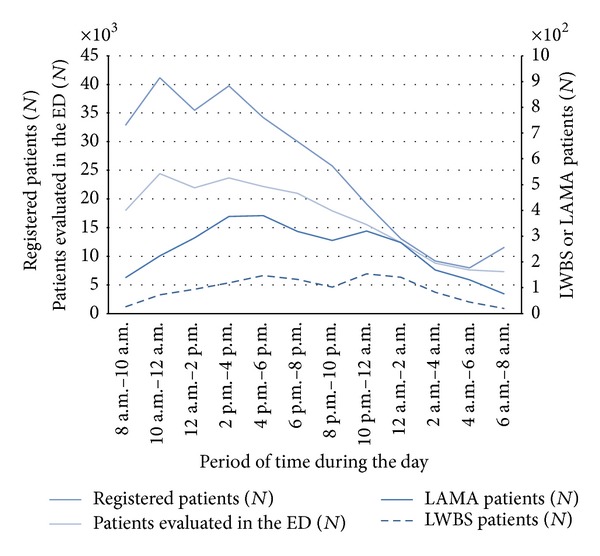
Repartition of the patients according to their time of arrival.

The repartitions of the cases according to the time of arrival show similar circadian distributions with an increase in the number of LWBS and LAMA patients during the [2 p.m.–8 p.m.] and [10 p.m.–2 a.m.] periods. These results were not related to the numbers of patients triaged or admitted in the ED but rather demonstrated a shift between time arrival in the ED and time of leaving (Figure 2).

Among the LWBS patients, the median time spent in the ED was 102 minutes (IQR: 62–156). The four most common initial reasons for consultation were related to orthopedic/trauma complaints (36.3%), abdominal pain or digestive complaints (13.6%), ear-nose-throat symptoms (9.2%), and neurological/neurosurgical complaints (7.1%). The acuity levels were moderate or low, with only one patient identified as requiring “immediate care” according to the triage level (a transient ischemic attack).

For LAMA patients, the median time before leaving was 226 minutes (IQR: 130–373). The most frequent reasons for leaving against medical advice were related to the perceived excessive length of stay (39.8%), a refusal of the proposed diagnostic or therapeutic strategy (35.2%), familial reasons (7.6%) or professional commitments (3.2%), and a spontaneous resolution of symptoms (6.2%).

## 4. Discussion

This study was aimed at evaluating the question of patients leaving without being seen or against medical advice over a six-year period in an academic teaching hospital ED.

In our study, the proportion of LWBS and LAMA patients are 0.4% and 0.9%, respectively. These rates remained unchanged over the six-year period.

Rates of LWBS and LAMA are used as indirect quality indicators in many healthcare systems and are therefore regularly monitored in order to evaluate the impact of overcrowding or resource allocation in the ED [1, 8, 34, 35]. Our rates are in the lower range of the results reported by other emergency services [21, 33, 36]. A slight but nonsignificant diminution in the rates of both LWBS and LAMA is noticeable since 2008. This trend may be secondary to a new internal organization of the ED, with the implementation of a “fast-track” strategy for minor trauma in 2008. The simultaneous diminution in the number of LWBS seeking medical consult for “orthopedic/trauma complaints” tends to corroborate this hypothesis. The design of our study does not allow us to fully confirm this hypothesis, but previous publications have demonstrated the same results [21, 37].

The rates of LWBS or LAMA are the highest among the 20- to 49-year-old patients, with a slight predominance of male patients. These findings are in line with the available literature [1, 2, 8, 13, 28, 35]. The acuity levels of LWBS patients are moderate to low, and the reasons for consultation mostly referred to trauma, abdominal pain, and ENT or neurological problems. The estimated median time before leaving is 102 minutes, a delay rarely reported in the literature but comparable to previous results in a Canadian study [28].

Our results are also in accordance with previous publications, showing that LWBS patients have a wide range of complaints, with musculoskeletal and gastrointestinal complaints ranked first, and a low acuity level [12, 29, 31]. Unlike other studies, complaints related to wounds are infrequent in our study, possibly reflecting a low rate of stab wounds [31].

These results should not hide the fact that these patients are nevertheless at higher risk of morbidity, mortality, and hospital readmission in the following weeks [2, 36, 38]. In the literature, some patients leave the ER with a triage category that indicates a potentially severe condition (i.e., headache, chest pain, and asthma). This is supported by our data, with 4–10% of LWBS patients triaged as urgent. The reasons for leaving are sometimes a function of worsening of condition “too sick to wait,” or a failure of pain relief [5, 7, 11, 39]. Prospective studies have shown that the readmission rate of these patients to the ED within 48 hours is between 15% and 20%, with a significant number of hospitalizations (2–10%) and some deaths [8, 40–42]. This point is in relation with a higher proportion of low-income and poorly insured patients [6, 15, 33, 38]. Both sets of patients are usually young men without insurance or a sociomedical support; alcohol or drug abuse is frequently also present [17, 19, 20, 29, 36, 38]. Therefore, on a healthcare point of view, the LWBS and LAMA patients should be seen as real “missed care opportunities” for the ED [2, 28].

The median time before leaving is higher for LAMA patients, about four hours. For these patients, the most common reason for leaving is related to the length of stay in the ER perceived as excessive. In most cases, prolonged waiting time and ER overcrowding are the main reasons for leaving [5, 10, 33, 43]. Other reasons cited in the literature include spontaneously improved symptoms, a conflict with the treatment team, or work or family commitments [5, 10, 14, 43]. Direct links between the waiting times and the proportion of patients who leave the ER have been shown many times (Table 3) and are often used as indicators of the quality of the ER in the USA [3, 24–26, 30, 35, 41, 44]. High rates of LWBS or LAMA are typically seen in urban public hospitals and teaching hospitals [33, 34]. In our study, the distribution of times of arrival was similar in LWBS and LAMA, with a slight increase in the afternoon and in the evening. The repartition of the cases during the day shows no clear correlation with the total number of patients triaged to or admitted in the ED. This result is in contradiction with other studies [28, 30]. Nevertheless, this fact is not surprising, as these indicators are not well correlated with the waiting times and occupation rate of the ED. A better indicator, not provided in our study, would be the mean number of patients present at a given time in the waiting room or in the ED ward or a composite indicator of overcrowding [26, 27].

Medicolegal consequences for the ED are for the moment not well-addressed in the literature. They may involve the entire hospital wards, inpatients bed-access unavailability being a major cause of ED overcrowding [19, 45].

## 5. Limitations

Our study is subject to several limitations. It was performed on retrospective data from one large urban, academic hospital only. The forms were however filled at the time patients left the ED. Selection bias could not be totally ruled out, with situations of patients, LWBS or LAMA, but not resulting to a report. The definition of LWBS may vary according to the organization of the hospital and of the ED [32]. In our study, we have no dedicated system to identify LWBS patients before being administratively registered. Regarding patients leaving before being evaluated by the TN, a lack of data cannot be ruled out, and the ED tracking system does not allow full identification of these patients. In comparison with previous publications, our results are in the lower range of the LWBS rates (Table 3). Nevertheless, because of the aforementioned limitations, our study may underestimate the real rate of LWBS [28, 29]. Finally, the monocentric nature of the study limits the interpretation and the extrapolation of our results to other settings.

## 6. Conclusion

The rates of LWBS and LAMA patients were low and remain unchanged over the six-year period. The patients shared similar characteristics and reasons for LWBS or LAMA were largely related to the length of stay and waiting times, therefore indirectly reflecting ED organizational conditions.

## Figures and Tables

**Table 1:** Left without being seen (LWBS) patients.

	2005	2006	2007	2008	2009	2010
Patients at triage (*n*)	47 974	50 384	48 562	51 557	55 276	53 963
Male/female ratio	1.1	1.1	1.1	1.1	1.1	1.1
Age: mean (range)	48.6 (16–104)	46.1 (16–104)	46.0 (16–106)	45.5 (16–107)	45.1 (16–108)	46.5 (16–109)

LWBS *n* (%)	171 (0.4%)	258 (0.5%)	244 (0.5%)	162 (0.3%)	163 (0.3%)	159 (0.3%)
Male/female ratio	1.3	1.3	1.3	1.3	0.8	1.4
Age: mean (range)	38.1 (17–82)	38.4 (16–95)	38.2 (16–86)	40.2 (16–90)	36.1 (16–86)	39.9 (18–89)
STS level acuity *n* (%):						
1	0 (0.0%)	0 (0.0%)	1* (0.4%)	0 (0.0%)	0 (0.0%)	0 (0.0%)
2	9 (5.3%)	11 (4.2%)	13 (5.3%)	8 (4.9%)	15 (9.2%)	15 (9.4%)
3 or more	161 (94.1%)	230 (88.8%)	200 (81.9%)	143 (88.3%)	147 (90.1%)	144 (90.6%)
Missing values	1 (0.6%)	17 (6.5%)	30 (12.3%)	11 (6.8%)	1 (0.6%)	0 (0.0%)
Complaints *n* (%)						
Traumatology/orthopaedic	84 (49.1)	115 (44.6)	106 (43.4)	51 (31.5)	40 (24.5)	24 (15.1)
Abdominal pain/digestive	14 (8.2)	33 (12.7)	30 (12.3)	29 (17.9)	26 (15.9)	26 (16.4)
ENT	14 (8.2)	17 (6.6)	19 (7.8)	24 (14.8)	14 (8.5)	19 (11.9)
Neurology/neurosurgery	8 (4.7)	19 (7.3)	16 (6.6)	7 (4.3)	17 (10.4)	16 (10.0)
Pulmonary	6 (3.5)	16 (6.2)	8 (3.8)	4 (2.5)	6 (3.7)	6 (3.8)
Cardiovascular	8 (4.7)	10 (3.8)	12 (4.9)	6 (3.7)	8 (4.9)	8 (5.0)
Infectious	11 (6.4)	13 (5.0)	7 (2.9)	5 (3.1)	7 (4.3)	4 (2.5)
Urology	11 (6.4)	13 (5.0)	7 (2.9)	6 (3.7)	6 (3.7)	6 (3.8)
Alcohol/toxic	3 (1.7)	5 (1.9)	8 (3.3)	8 (4.9)	5 (3.1)	8 (5.0)
Endocrinology, diabetes	0 (0.0)	0 (0.0)	4 (1.6)	1 (0.6)	0 (0.0)	4 (2.5)
Psychiatry	0 (0.0)	3 (1.2)	2 (0.8)	2 (1.2)	3 (1.8)	0 (0.0)
General	4 (2.3)	4 (1.6)	14 (5.7)	14 (8.6)	11 (6.7)	15 (9.4)
Allergy/dermatology	8 (4.7)	10 (3.8)	7 (2.9)	4 (2.4)	7 (4.3)	8 (5.0)
Wounds	0 (0.0)	0 (0.0)	4 (1.6)	1 (0.6)	13 (7.9)	15 (9.4)
Time before leaving (min)						
Median	116	109.5	118	96	82	94
IQR	[69.5–158]	[65.0–162.8]	[72.5–175.0]	[56.3–148.5]	[45.5–130.5]	[63.0–145.0]

STS: Swiss triage scale, LWBS: left without being seen, ENT: ear-nose-throat, and IQR: interquartile range.

*One case of transient ischemic stroke, with spontaneous complete recovery.

**Table 2:** Left against medical advice (LAMA) patients.

	2005	2006	2007	2008	2009	2010
Patients admitted in the ED	30 487	32 465	32 335	34 420	36 140	36 704
Male/female ratio	1.1	1.1	1.1	1.1	1.1	1.2
Age: mean (range)	49 (16–104)	48.7 (16–104)	49.0 (16–106)	48.8 (16–107)	49 (16–108)	49.8 (16–109)

LAMA *n* (%)	271 (0.9%)	317 (1.0%)	370 (1.1%)	322 (0.9%)	282 (0.8%)	291 (0.8%)
Male/female ratio	1.4	1.2	1.2	1.1	1.2	1.3
Age: mean (range)	41.2 (16–90)	40.6 (16–88)	41.3 (16–95)	43.1 (17–92)	41.7 (17–91)	43.2 (17–95)
Reasons for leaving *n* (%)						
waiting time*	115 (42.4)	133 (41.9)	164 (44.3)	127 (39.4)	121 (42.9)	77 (26.4)
refusal of diagnostic or therapeutic proposition	109 (40.2)	120 (37.9)	109 (29.5)	114 (35.4)	79 (28.0)	122 (41.9)
spontaneous positive evolution	8 (2.9)	12 (3.8)	30 (8.1)	23 (7.1)	18 (6.4)	25 (8.5)
familial concern	13 (4.8)	17 (5.3)	29 (7.8)	27 (8.3)	25 (8.8)	31 (10.6)
unknown	12 (4.4)	20 (6.3)	20 (5.4)	18 (5.6)	11 (3.9)	14 (4.8)
economic or insurance	3 (1.1)	6 (1.9)	6 (1.6)	2 (0.6)	7 (2.5)	12 (4.1)
professional concern	11 (4.1)	8 (2.5)	12 (3.2)	11 (3.4)	21 (7.4)	10 (3.5)
missing data	0 (0.0)	1 (0.3)	0 (0.0)	0 (0.0)	0 (0.0)	0 (0.0)
Time before leaving (min)						
Median	211	203	243	254	219	244
IQR	[114.5–313.0]	[114.0–335.0]	[136.5–359.0]	[144.5–424.8]	[116.1–395.0]	[154.0–407.5]

STS: Swiss Triage Scale, LAMA: left against medical advice, IQR: interquartile range.

*Waiting for radiology imaging, biological exams, specialized medical evaluation.

**Table 3:** Review of LWBS rates and associated patients and ED characteristics.

Studies	Ref.	Countries	Year	Rate	Setting	Patients factors	ED factors
Fry et al., 2003	[7]	Australia	2000–2002	7.9%	Tertiary hospital	Nonurgent conditions	Waiting time
Arendt et al., 2003	[5]	USA	2001	0.84%	Small Hospital	NA	NA
Polevoi et al., 2005	[24]	USA	2001	1.8%	University hospital	NA	Overcrowding EP training
Goodacre and Webster, 2005	[13]	UK	2001	7.2%	Non-univ. tertiary hospital (adults)	Young, male Low acuity level	Waiting time
Goldman et al., 2005	[25]	Canada	2002	3%	Pediatric	Nonurgent conditions	Overcrowding Waiting time
Monzon et al., 2005	[11]	Canada	2003	3.6%	University hospital	Lack of regular physician	Waiting time
Weiss et al., 2005	[26]	USA	2003	14.9%	University hospital	NA	Overcrowding
Locker and Mason, 2005	[27]	England	2004	3.9%	83 EDs	NA	NA
Rowe et al., 2006	[12]	Canada	2002 + 2003	4.5%	University hospital (adults and pediatrics)	Low acuity level	Overcrowding
Baibergenova et al., 2006	[28]	Canada	2006	3.1%	163 EDs	Aged 15–35 Low acuity level	High volume of patient Length of stay
Ding et al., 2006	[6]	USA	2004	6.4%	University hospital (adults)	Aged 18–24 Low acuity level Uninsured or Medicaid	NA
Sun et al., 2007	[1]	USA	1995–2002	1.4%	National survey	Young, nonwhite, and urban Uninsured Nonurgent conditions	NA
Mohsin et al., 2007	[8]	Australia	2003	8.6%	Non-univ. tertiary hospital	Young Nonurgent conditions	Overcrowding Waiting time
Hall and Jelinek, 2007	[29]	Australia	2000–2003	4.1%	7, public hospital	Low acuity level Young, male	NA
Ding et al., 2007	[2]	USA	2004-2005	8.8%	University hospital (adults)	NA	NA
Asaro et al., 2007	[30]	USA	2004–2006	8.1%	University hospital	NA	Time of arrival Overcrowding
Gilligan et al., 2009	[31]	Ireland	NA	7.5%	University hospital	Young, male Low acuity level Arriving at night or at week-end	NA
Pham et al., 2009	[32]	USA	1998–2006	1.7%	National survey	Aged 18–65 Uninsured or Medicaid Low acuity level	Metropolitan areas Teaching hospital
Hsia et al., 2011	[33]	USA	2007	2.6%	262 EDs	Poorly insured Lower income	County owner Trauma center Teaching hospital
Grosgurin et al., 2013	[22]	Switzerland	2008	4.2%	University hospital (adults)	Male, unemployed, and low acuity level	NA

Non-univ.: Non-university hospital.

## References

[1] Sun BC, Binstadt ES, Pelletier A, Camargo CA (2007). Characteristics and temporal trends of “left before being seen” visits in US emergency departments, 1995–2002. *Journal of Emergency Medicine*.

[2] Ding R, Jung JJ, Kirsch TD, Levy F, McCarthy ML (2007). Uncompleted emergency department care: patients who leave against medical advice. *Academic Emergency Medicine*.

[3] Welch S, Augustine J, Camargo CA, Reese C (2006). Emergency department performance measures and benchmarking summit. *Academic Emergency Medicine*.

[4] Bragulat E, Ortega M, Miró O (2008). Increasing number of patients who leave the ED without being seen. *Emergency Medicine Journal*.

[5] Arendt KW, Sadosty AT, Weaver AL, Brent CR, Boie ET (2003). The left-without-being-seen patients: what would keep them from leaving?. *Annals of Emergency Medicine*.

[6] Ding R, McCarthy ML, Li G, Kirsch TD, Jung JJ, Kelen GD (2006). Patients who leave without being seen: their characteristics and history of emergency department use. *Annals of Emergency Medicine*.

[7] Fry M, Thompson J, Chan A (2003). Patients regularly leave emergency departments before medical assessment: a study of did not wait patients, medical profiles and outcome characteristics. *The American Journal of Nursing*.

[8] Mohsin M, Forero R, Ieraci S, Bauman AE, Young L, Santiano N (2007). A population follow-up study patients who left an emergency department without being seen by a medical officer. *Emergency Medicine Journal*.

[9] Mohsin M, Bauman A, Ieraci S (1998). Is there equity in emergency medical care? Waiting times and walk-outs in South Western Sydney hospital emergency departments. *Australian Health Review*.

[10] Fernandes CMB, Daya MR, Barry S, Palmer N (1994). Emergency department patients who leave without seeing a physician: the Toronto Hospital experience. *Annals of Emergency Medicine*.

[11] Monzon J, Friedman SM, Clarke C, Arenovich T (2005). Patients who leave the emergency department without being seen by a physician: a control-matched study. *Canadian Journal of Emergency Medicine*.

[12] Rowe BH, Channan P, Bullard M (2006). Characteristics of patients who leave emergency departments without being seen. *Academic Emergency Medicine*.

[13] Goodacre S, Webster A (2005). Who waits longest in the emergency department and who leaves without being seen?. *Emergency Medicine Journal*.

[14] Khanna R, Chaudhry MA, Prescott M (1999). Emergency department patients who leave the department without being seen by a doctor. *European Journal of Emergency Medicine*.

[15] Franks P, Meldrum S, Fiscella K (2006). Discharges against medical advice: are race/ethnicity predictors?. *Journal of General Internal Medicine*.

[16] McCaig LF, Burt CW (2005). *National Hospital Ambulatory Medical Care Survey: 2003 Emergency Department Summary*.

[17] Jeremiah J, O’Sullivan P, Stein MD (1995). Who leaves against medical advice?. *Journal of General Internal Medicine*.

[18] Pennycook AG, McNaughton G, Hogg F (1992). Irregular discharge against medical advice from the accident and emergency department—a cause for concern. *Archives of Emergency Medicine*.

[19] Henson VL, Vickery DS (2005). Patient self discharge from the emergency department: who is at risk?. *Emergency Medicine Journal*.

[20] Dubow D, Propp DA, Narasimhan K (1992). Emergency department discharges against medical advice. *Journal of Emergency Medicine*.

[21] Kennedy M, MacBean CE, Brand C, Sundararajan V, McD Taylor D (2008). Review article: leaving the emergency department without being seen. *Emergency Medicine Australasia*.

[22] Grosgurin O, Cramer B, Schaller M, Sarasin F, Rutschmann OT (2013). Patients leaving the emergency department without being seen by a physician: a retrospective database analysis. *Swiss Medical Weekly*.

[23] Rutschmann OT, Kossovsky M, Geissbühler A (2006). Interactive triage simulator revealed important variability in both process and outcome of emergency triage. *Journal of Clinical Epidemiology*.

[24] Polevoi SK, Quinn JV, Kramer NR (2005). Factors associated with patients who leave without being seen. *Academic Emergency Medicine*.

[25] Goldman RD, Macpherson A, Schuh S, Mulligan C, Pirie J (2005). Patients who leave the pediatric emergency department without being seen: a case-control study. *Canadian Medical Association Journal*.

[26] Weiss SJ, Ernst AA, Derlet R, King R, Bair A, Nick TG (2005). Relationship between the national ED overcrowding Scale and the number of patients who leave without being seen in an academic ED. *The American Journal of Emergency Medicine*.

[27] Locker TE, Mason SM (2005). Analysis of the distribution of time that patients spend in emergency departments. *British Medical Journal*.

[28] Baibergenova A, Leeb K, Jokovic A, Gushue S (2006). Missed opportunity: patients who leave the emergency departments without being seen. *Healthcare Policy*.

[29] Hall J, Jelinek GA (2007). Characteristics and outcomes of patients who “did not wait” after attending Perth public hospital emergency departments, 2000–2003. *Medical Journal of Australia*.

[30] Asaro PV, Lewis LM, Boxerman SB (2007). Emergency department overcrowding: analysis of the factors of renege rate. *Academic Emergency Medicine*.

[31] Gilligan P, Joseph D, Winder S (2009). DNW: “did not wait” or “demographic needing work”: a study of the profile of patients who did not wait to be seen in an Irish emergency department. *Emergency Medicine Journal*.

[32] Pham JC, Ho GK, Hill PM, McCarthy ML, Pronovost PJ (2009). National study of patient, visit, and hospital characteristics associated with leaving an emergency department without being seen: predicting LWBS. *Academic Emergency Medicine*.

[33] Hsia RY, Asch SM, Weiss RE (2011). Hospital determinants of emergency department left without being seen rates. *Annals of Emergency Medicine*.

[34] Bolton L, Robinson K, Burns K (2006). Factors that correlate with patients leaving an emergency department without being seen. *Annals of Emergency Medicine*.

[35] Asaro P, Lewis L, Boxerman S (2005). Logistic regression analysis of the determinants of renege likelihood in a large urban emergency department. *Academic Emergency Medicine*.

[36] Garland A, Ramsey C, Fransoo R (2013). Rates of readmission and death associated with leaving hospital against medical advice: a population-based study. *Canadian Medical Association Journal*.

[37] Chan TC, Killeen JP, Kelly D, Guss DA (2005). Impact of rapid entry and accelerated care at triage on reducing emergency department patient wait times, lengths of stay, and rate of left without being seen. *Annals of Emergency Medicine*.

[38] Hwang SW, Li J, Gupta R, Chien V, Martin RE (2003). What happens to patients who leave hospital against medical advice?. *Canadian Medical Association Journal*.

[39] Baker DW, Stevens CD, Brook RH (1991). Patients who leave a public hospital emergency department without being seen by a physician: causes and consequences. *Journal of the American Medical Association*.

[40] Welch S (2005). Matchmaking: emergency medicine benchmarking basics. *Emergency Medicine News*.

[41] Hobbs D, Kunzman SC, Tandberg D, Sklar D (2000). Hospital factors associated with emergency center patients leaving without being seen. *The American Journal of Emergency Medicine*.

[42] Torres V, Brunett P, Fu R (2007). Leaving without being seen. Boarding, bored, or both?. *Academic Emergency Medicine*.

[43] Rowe B, Channan P, Bullard M (2003). Reasons why patients leave without being seen from the emergency department. *Academic Emergency Medicine*.

[44] Stock LM, Bradley GE, Lewis RJ, Baker DW, Sipsey J, Stevens CD (1994). Patients who leave emergency departments without being seen by a physician: magnitude of the problem in Los Angeles County. *Annals of Emergency Medicine*.

[45] Richardson DB (2002). The access-block effect: relationship between delay to reaching an inpatient bed and inpatient length of stay. *Medical Journal of Australia*.

